# An Account of an Epidemic of Scarlatina, with Pustular Eruptions

**Published:** 1840-01-18

**Authors:** Richard A. Sale

**Affiliations:** Bedford County, Va.


### AN ACCOUNT OF AN EPIDEMIC OF SCARLATINA, *with Pustular Eruptions.* By RICHARD A. SALE, M. D., of Bedford County, Va.

To the Editors of the Medical Examiner.

The section of country in which the present epidemic made its appearance, lies immediately at the base of the Blue Ridge. It is narrow, and is surrounded by mountains in all directions, except the eastern, which serves for the passage of the only stream of water it possesses, which is remarkable for its transparency and its purity. Hitherto autumnal fevers and malarious diseases have not been known. The epidemic in question presented all the phenomena usually observed in the severer grades of scarlatina, with unusual and additional phenomena. In addition to the rash generally observed in scarlet fever, there was superadded a pustular eruption, which resembled considerably the eruption of varicella and vaccina, more particularly the latter, varying in size from the eruption of varicella to that of a well defined vaccine pustule. This eruption did not make its appearance as soon as the scarlet rash, but generally at the declension of the pyrexia, and a little antecedent to desquamation. It was more frequent in cases in which enteritis was a prominent symptom from the commencement, and in which typhoid symptoms early supervened. The cases in which this eruption appeared, were more unmanageable than the others. The disease seemed to confine its desolating effects principally to children, whilst adults had it only in the simple form. The anginose affection in children was extremely severe and distressing. In many instances, if they passed safely through the immediate effects of the disease, they were left with enormous abscesses on the exterior of the neck, and sometimes in the parotid glands. Not unfrequently, and during the greatest pyrexia, the joints of the upper and lower extremities became exceedingly painful, and very much enlarged, resembling acute rheumatism.

With respect to the treatment, I will merely add that it was moderately antiphlogistic, and conducted with a due regard to the early stage in which collapse took place. Venesection and purgatives were not used. The former would have been hazardous, whilst the latter was not indicated, the bowels being in an irritated condition from the beginning.

Mercurial alteratives, combined with ipecacuanha, or the pulvis Doveri, seemed to answer a good purpose in my hands. Revulsives to the throat were beneficial, if applied early. Gargles seemed to be more beneficial as detergents than as medicaments.

*Bedford county, Va., Jan. 1st,* 1840.

